# An *in vitro* liver model - assessing oxidative stress and genotoxicity following exposure of hepatocytes to a panel of engineered nanomaterials

**DOI:** 10.1186/1743-8977-9-28

**Published:** 2012-07-19

**Authors:** Ali Kermanizadeh, Birgit K Gaiser, Gary R Hutchison, Vicki Stone

**Affiliations:** 1 Heriot-Watt University, School of Life Sciences, Nanosafety Research Group, Edinburgh, EH14 4AS, UK; 2 Edinburgh Napier University, School of Life, Sport and Social Sciences, Sighthill Campus, Sighthill Court, Edinburgh, EH11 4BN, UK

**Keywords:** Liver, Nanomaterials, Oxidative stress, Antioxidant, Genotoxicity

## Abstract

**Background:**

Following exposure via inhalation, intratracheal instillation or ingestion some nanomaterials (NM) have been shown to translocate to the liver. Since oxidative stress has been implicated as a possible mechanism for NM toxicity this study aimed to investigate the effects of various materials (five titanium dioxide (TiO_2_), two zinc oxide (ZnO), two multi-walled carbon nanotubes (MWCNT) and one silver (Ag) NM) on oxidative responses of C3A cell line as a model for potential detrimental properties of nanomaterials on the liver.

**Results:**

We noted a dose dependant decrease in the cellular glutathione content following exposure of the C3A cells to Ag, the ZnO and the MWCNTs. Intracellular ROS levels were also measured and shown to increase significantly following exposure of the C3A to the low toxicity NMs (MWCNT and TiO_2_). The antioxidant Trolox in part prevented the detrimental effect of NMs on cell viability, and decreased the NM induced IL8 production after exposure to all but the Ag particulate. Following 4 hr exposure of the C3A cells to sub-lethal levels of the NMs, the largest amount of DNA damage was induced by two of the TiO_2_ samples (7 nm and the positively charged 10 nm particles).

**Conclusions:**

All ten NMs exhibited effects on the hepatocyte cell line that were at least in part ROS/oxidative stress mediated. These effects included mild genotoxicity and IL8 production for all NM except the Ag possibly due to its highly cytotoxic nature.

## Background

As the field of nanotechnology develops, there are now over 1300 consumer products on the market with a claim to contain elements of nanotechnology [1]. The potential for public and occupational exposure is therefore likely to increase, and so there is an urgent necessity to consider the possibility of any detrimental health consequences with this increased exposure to nanomaterials. This is achieved in the form of a critical risk assessment conducted as part of a large consortium (FP7 project – ENPRA – Risk Assessment of Engineered Nanoparticle). The risk is assessed based upon the level of exposure to the manufactured NM, toxicity of the particle in question, route of exposure and the persistence in the organism of the particular material. Hence it is crucial to identify the hazards associated with NM exposure both *in vitro* and *in vivo*, consequently assembling a knowledge base of the human health effects associated with NM exposure [2]. Engineered nanomaterials are manufactured from a diverse group of substances each with an array of unique physicochemical characteristics, hence a varied range of materials need to be evaluated for a comprehensive toxicity profile allowing for a structure activity relationship to be generated. It is likely that NMs will differ in the levels of toxicity induced and the mechanism by which they exert these adverse effects. Therefore we investigated a panel of ten widely used nanomaterials (five different TiO_2_, two MWCNTs, two ZnO and one Ag).

The lungs and the gastrointestinal tract are in constant contact with the external environment so it is not surprising to find these systems being primary exposure sites for NMs [3,4]. It is believed that NMs administered via the ingestion, inhalation or intravenous injection might eventually reach secondary tissues, one of which is the liver [5,6].

The liver is the metabolic centre of the body [6]. It has a crucial role in metabolic homeostasis, as it is responsible for the storage, synthesis, metabolism and re-distribution of carbohydrates, fats and vitamins. It also produces large numbers of serum proteins and an array of enzymes and cytokines [7]. The liver receives and accumulates materials at much higher volumes compared to other organs and alongside the kidneys might be responsible for the clearance of NMs from the blood [6,8,9]. Previous studies have shown that the uptake and translocation of TiO_2_ NMs following intratracheal instillation has resulted in accumulation of nanomaterials within the liver [8-10].

There is an abundant body of evidence suggesting the involvement of oxidative stress in the pathogenesis of various disorders and diseases. Reactive oxygen species (ROS) and other free radicals are critical intermediates in the normal physiology and pathophysiology of the liver [5]. Oxygen species are important in the creation of oxidative stimuli required for normal physiologic homeostasis of hepatocytes, as well as playing a role in gene expression [5]. Since ROS are ubiquitous in the normal physiology of so many processes, it is not surprising that when excess ROS are produced, some normal functions of healthy cells are affected.

To combat excess ROS, cells utilise antioxidants. If the equilibrium between ROS generation and the antioxidant defence within a cell is disrupted it may result in oxidative stress [11]. Common antioxidants in hepatocytes include glutathione (GSH), glutathione peroxidase, superoxide dismutase (SOD), hemeoxygenase (HO) and peroxidases [12]. Glutathione is a ubiquitous tri-peptide which primarily functions to react with hydrogen peroxide utilising glutathione peroxidise to create glutathione disulfide (GSSG). GSH also scavenges other ROS molecules and prevents oxidation of protein sulfhydryl groups [5].

The effects of oxidative stress are usually dependent upon the size of these changes, with a cell being able to overcome small perturbations and regain its original state. However, long lasting or severe oxidative stress can cause cell damage and death. Even moderate oxidative stress can trigger apoptosis, while more intense stress may cause necrosis [13]. Mild ROS/oxidative stress can activate cells via redox sensitive transcription factors (i.e. NFκβ) leading to elevated gene expression of pro-inflammatory mediators [14], while severe ROS insult can lead to genotoxicity [15,16].

The nanomaterials in this study were chosen due to their varied physicochemical characteristics, their relevance to the OECD (Organisation for Economic Co-operation and Development) sponsored programme and their frequent use in various industries. In this study intracellular levels of glutathione were measured in human hepatoblastoma C3A cell line as well as intracellular ROS using the 2′ 7′ – dichlorofluorescin diacetate (DCFH-DA) assay. Antioxidants were used to investigate the role of ROS in the responses observed. Furthermore, the short term genotoxic properties of the panel of materials was investigated utilising the widely acknowledged comet assay.

## Methods

### Nanomaterials

Nanomaterials were purchased as stated: NM 101 (Hombikat UV100; rutile with minor anatase; 7 nm), NM 110 (BASF Z-Cote; zinkite, uncoated, 100 nm), NM 111 (BASF Z-Cote; zinkite coated with triethoxycaprylylsilane, 130 nm), NM 300 (RAS GmbH; Ag capped with polyoxylaurat Tween 20 - <20 nm), NM 400 (Nanocyl; entangled MWCNT, diameter 30 nm, length 5 μm), NM 402 (Arkema Graphistrength C100; entangled MWCNT, diameter 30 nm, length 20 μm). The above mentioned nanomaterials were sub-sampled under Good Laboratory Practice conditions and preserved under argon in the dark until use. These NMs were received from the European Commission Joint Research Centre (Ispra, Italy). The NRCWE samples were procured by the National Research Centre for the Working Environment. Sub-sampling was completed into 20 ml Scint-Burk glass pp-lock with Alu-Foil (WHEA986581; Wheaton Industries Inc.) after pooling and mixing of the material. NRCWE 001, TiO_2_ rutile 10 nm was purchased from NanoAmor (Houston, USA) and also used for production of NRCWE 002 (TiO_2_ rutile 10 nm with positive charge) and NRCWE 003 (TiO_2_ rutile 10 nm with negative charge) using the procedures described previously [17]. NRCWE 004 (TiO_2_ rutile 94 nm) was purchased from NaBond. A list of the main physical and chemical properties of the panel NMs has been reproduced from previously described work [17] (Table 1).

**Table 1:** **Physicochemical characteristics of engineered nanomaterials investigated - reproduced from Kermanizadeh, *et al.***[17]

**ENM code**	**ENM type**	**Phase**	**XRD Size[nm]**	**TEM Size**	**Primary characteristics by TEM analysis**	**Surface area (BET)[m**^**2**^**/g]**	**Known coating**	**Size in MEM (DLS) Ψ**
NM101	TiO_2_	Anatase^€^	9	4-8/50-100	Two structures found; type 1 show agglomerates in the 50–1500 nm range	322	none	185, 742
NM110	ZnO	Zincite	70 to > 100	20-250/50-350	Mainly 2 euhedral morphologies: 1) aspect ratio close to 1 (20–250 nm range and few particles of approx. 400 nm)2) ratio 2 to 7.5 (50–350 nm). Minor amounts of particles with irregular morphologies observed.	14	none	306
NM111	ZnO	Zincite	58-93	20-200/10-450	As NM110, but with different size distributions. 1) particles with aspect ratio close to 1 (~90% in the 20–200 nm range); 2) particles with aspect ratio 2 to 8.5 (~90% in the 10–450 nm ratio).	18	Trie-othoxy-capry-lsilane 130	313
NM300	Ag	Ag_m_	7^$^14^£^ < 18/15/> 100^#^	8-47 (av.: 17.5)	Mainly euhedral NP; minor fractions have either elongated (aspect ratio up to ~ 5) or sub-spherical morphology.	NA	none	12, 28, 114
NM400	MWCNT	-	-	D: 5–35L: 700-3000	Irregular entangled kinked and mostly bent MWCNT (10–20 walls). Some CNTs were capped and some cases multiple caps were found due to overgrowth. Fe/Co catalysts (6–9 nm, average 7.5 nm) were found inside the tubes.	298	none	*
NM402	MWCNT	-	-	D: 6–20L: 700-4000	Entangled irregular, mostly bent MWCNT (6–14 walls). Some tubes were capped by unknown material. Some nano-onions (5–10 nm) and amorphous carbon structures mixed with Fe (5–20 nm). Residual catalyst was observed. Individual catalyst particles up to 150 nm were also detected.	225	none	*
NRCWE001	TiO_2_	Rutile^§^	10	80-400	Irregular euhedral particles detected by TEM.	99	none	203
NRCWE002	TiO_2_	Rutile	10	80-400	Irregular euhedral particles detected by TEM.	84	Positive charged	287
NRCWE003	TiO_2_	Rutile	10	80-400	Irregular euhedral particles detected by TEM.	84	Negative charged	240, 1487
NRCWE004	TiO_2_	Rutile	App. 100	1-4/10-100/100-200/1000-2000	Five different particle types were identified: 1) irregular spheres, 1–4 nm (av. Diameter); 2) irregular euhedral particles, 10–100 nm (longest dimension); 3) fractal-like structures in long chains, 100–200 nm (longest dimension); 4) big irregular polyhedral particles, 1-2 μm (longest dimension); 5) large irregular particles with jagged boundaries, 1–2 μm (longest dimension).			339

^€^ 1 percent rutile found in one of two samples analyzed.

^$^ wet XRD in capillary tube.

^£^ dried samples.

^#^ sample with deposits.

^§^ ca. 6% anatase was observed in one of two samples analyzed.

* Not detectable by DLS due to the very large aspect ratio.

Ψ Intensity based size average in biological media after 15 mins.

Abbreviations: *D* Diamter, *DLS* Dynamic Light Scattering, *ENM* Engineered nanomaterial, *L* Length, *XRD* X ray diffraction.

### Cell culture and treatment with nanomaterials

The human hepatoblastoma C3A cell line was obtained from the American Type Culture Collection (ATCC, USA). The cells were maintained in Minimum Essential Medium Eagle (MEM) with 10% FCS, 2 mM L-glutamine, 100 U/ml Penicillin/Streptomycin, 1 mM sodium pyruvate, and 1% non essential amino acids (here after termed complete medium), at 37°C and 5% CO_2_. All experiments were conducted using cells between passage 7 and 25.

The Ag NMs were supplied in de-ionised water with stabilizing agent (7% ammonium nitrate, 4% each of Polyoxyethylene Glycerol Trioleate and 4% Tween 20). All other NMs were supplied as a dry powder form and dispersed utilising MilliQ de-ionised water with 2% fecal calf serum (FCS – Sigma B9433), with the exception of the coated ZnO materials, which were wetted with 0.5% vol ethanol before the addition of the dispersion media. The nanomaterials were sonicated for 16 mins without pause following the protocol developed for ENPRA [18]. Following sonication, all samples were kept on ice until dilution in complete medium.

To examine the toxicity of nanomaterials to C3A cells, NM concentrations between 0.16 μg/cm^2^ and 80 μg/cm^2^ were used (equivalent to 0.5–256 μg/ml).

### Antioxidant pre-treatment

The C3A cells were seeded in 96 well plates at 10^4^ cells per well in 100 μl of the complete cell culture medium and incubated for 24 hr at 37°C and 5% CO_2_. The following day the cells were exposed to the nanomaterials or controls for a further 24 hr. In order to investigate the possible intervention of antioxidants on cytotoxicity, ROS or cytokine production from the C3A cells, they were pre-treated with 100 μM of Trolox (6-hydroxy-2,5,6,7,8-tetramethylchroman-2-carboxylic acid) in complete medium for 1 hr. The antioxidant containing medium was removed before the addition of the nanomaterials as described above.

### Measurement of total glutathione

The protocol is adapted from Senft *et al.*[19]. A 3 ml cell suspension of cultured cells (1×10^6^ cells per ml) was added to 6 well plates and incubated overnight at 37°C and 5% CO_2_. The cells were exposed to the NMs or equivalent control dispersant in complete C3A medium for 24 hr before being scraped into ice cold phosphate buffered saline and centrifuged (700 g for 2 mins). The cell pellet was re-suspended in ice-cold lysis buffer [19], mixed and incubated on ice for 10 mins before being centrifuged at 15000 g for 5 mins to generate lysates and protein pellets. Glutathione was quantified of the lysate by reaction of sulfhydryl groups with the fluorescent substrate *o*-phthaladehyde (OPT) using a fluorimeter with an excitation wavelength of 350 nm and emission wavelength of 420 nm.

The protocol was slightly modified to include measurements of total glutathione by reducing oxidised glutathione dimers (GSSG) by addition of 7 μl of 10 mM sodium dithionite to all samples and incubating at room temperature for 1 hr.

### DCFH-DA assay

C3A cells were seeded on a 96 well plate in complete phenol red free C3A medium (1×10^4^ cells per well) and incubated at 37°C, 5% CO_2_ for 24 hr. The cells were exposed to the NMs or equivalent control (hydrogen peroxide 100 μM – positive control) in complete medium for 24 hr.

DCFH-DA is air, light and temperature sensitive so great care was taken when preparing the final working concentration of 10 μM in 0.9% NaCl. Following incubation, cells were rinsed and 100 μl of DCFH-DA was added before the plates were incubated in the dark at room temperature for 1 hr. Cells were rinsed again and 200 μl of 90% DMSO in PBS was added and incubated on a shaker for 5 mins at room temperature. The plates were wrapped in foil to protect from light before being centrifuged for 2 mins at 250 g. This was followed by the measurement of 150 μl of supernatant in black 96 well plates at an excitation wavelength of 485 nm and emission wavelength of 520 nm.

### Detection of DNA strand breaks in C3A cells

The FPG (formamidopyrimidine [fapy] – DNA glycosylase) modified Comet assay was used to measure DNA strand breaks and specific oxidative DNA damage such as 7, 8-dihydro-8-oxoguanine, 8-oxoadenine, fapy-guanine etc., based on the method described by Speit *et al*. [20]. In this study the tail moment [21] was measured using an automatic image analyser (Comet Assay IV; Perceptive Instruments, UK) connected to a fluorescence microscope. Images were captured using a stingray (F-033B/C) black and white video camera.

After a 4 hr NM treatment (or positive control - 60 μM of H_2_O_2_), the C3A cells were rinsed twice with PBS and detached using trypsin before being suspended in 5 ml of culture medium. Cells were centrifuged for 10 mins at 250 g, 4°C and re-suspended at a concentration of 1.5 × 10^6^ cells/ml in complete medium. A 20 μl volume of calculated cell suspension was added to 240 μl of 0.5% low melting point agarose. Next, 125 μl of the mixture was added to pre-coated slides (1.5% agarose) in triplicate. Following 10 mins of solidification on ice, slides were lyzed overnight at 4°C in lysis buffer (2.5 M NaCl, 100 mM EDTA, 10 mM Tris-base, pH 10, containing 10% DMSO and 1% TritonX-100). The slides were washed three times for 5 mins with FPG-enzyme buffer (40 mM HEPES, 100 mM KCl, 0.5 mM EDTA, 0.2 mg/ml BSA - pH 8), covered with 100 μl of either buffer or FPG in buffer (1:30), sealed with a cover slip and incubated for 30 mins at 37°C. FPG cleaves DNA at locations of oxidation leading to a greater tail for cells exhibiting oxidative DNA damage [22]. All slides were then transferred into a black chilled electrophoresis tank. After alkaline unwinding (pH 13) for 20 mins, electrophoresis was performed for 15 mins at 270 mA, 24 V. Slides were neutralized three times for 5 mins using a neutralization buffer (0.4 M TrisBase, pH 7.5). Before analysis, slides were dried on air for 10 mins and stained with GelRed (2 in 10000, 40 μl per slide). A total of 50 cells were analyzed per slide per experiment.

We also investigated the long term genotoxic ability of Ag NM (NM 300). C3A cells were exposed to the NMs for 24 hr before being allowed to recover for 72 hr. The cells were then detached by treating with trypsin before being transferred to a new flask. After 48 hr the cells were treated with the Ag NMs for 24 hr, with the whole process being repeated for a period of 8 weeks.

### Statistical analysis

All data are expressed as mean ± standard error of the mean. For statistical analysis, the experimental results were compared to their corresponding control values using an ANOVA with Tukey’s multiple comparison. All statistical analysis was carried out utilising Minitab 15. A p value of < 0.05 was considered to be significant. All experiments were repeated a minimum of three times.

## Results

### Impact of the nanomaterials on depletion of GSH in C3A hepatocytes

**Figure 1:**
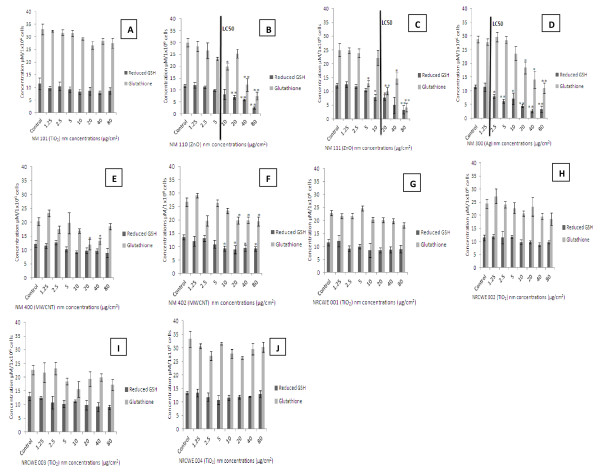
**Effects of NM exposure on reduced GSH and total glutathione levels in C3A cells.** The cells were exposed to cell medium (control), and increasing concentrations of selected NMs for 24 hr. Values represent mean ± SEM (n = 3), significance indicated by * = *p* < 0.05 and ** = *p* < 0.005 compared to the control. **A**) NM 101 **B**) NM 110 **C**) NM 111 **D**) NM 300 **E**) NM 400 **F**) NM 402 **G**) NRCWE 001 **H**) NRCWE 002 **I**) NRCWE 003 **J**) NRCWE 004. LC_50_ values are indicated for NMs where this value was measurable. For all other NMs the LC_50_ was not reached following exposure up to 80 μg/cm^2^ after 24 hr of incubation.

Analysis of the total glutathione contents of C3A cells revealed a dose dependant decrease compared to the control cells at 24 hr following exposure to five of the ten nanomaterials investigated. These NMs were NM 110 (ZnO uncoated), NM 111 (ZnO coated), NM 300 (Ag), NM 400 and NM 402 (2 MWCNTs) (Figure 1b, c, d, e and f). The three NMs previously shown to be the most cytotoxic to C3A cells as measured by the WST-1 assay (Ag NM 300, ZnO NM 110 and 111) also proved to induce relatively greater glutathione depletion than the other investigated NMs [17] (figure 1b, c and d - the LC_50_ is indicated).

### Measurement of intracellular ROS

**Figure 2:**
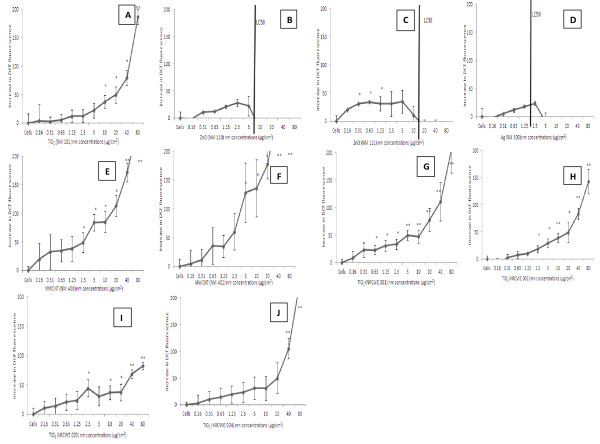
**Effects of increasing concentration of NMs on the oxidation of DCFH to DCF in the presence of C3A cells.** The C3A cells were exposed to cell medium (control) or NMs for 24 hr. Results are shown as mean fluorescence intensity minus corresponding control traces (± SEM) from three experiments (n = 3), significance indicated by * = *p* < 0.05 and ** = *p* < 0.005, when NM treatments are compared to the control. **A**) NM 101 **B**) NM 110 **C**) NM 111 **D**) NM 300 **E**) NM 400 **F**) NM 402 **G**) NRCWE 001 **H**) NRCWE 002 **I**) NRCWE 003 **J**) NRCWE 004. LC_50_ values are indicated for NMs where this value was measurable. For all other NMs the LC_50_ was not reached following exposure up to 80 μg/cm^2^ after a 24 hr incubation.

The 2′,7′-dichlorfluorescein-diacetate assay is based on the principle of DCFH being oxidized to fluorescent dichlorofluorescein (DCF) in the presence of intracellular ROS. We chose six different exposure time points (2, 4, 6, 8, 12 and 24 hr) and found the 6 hr exposure to be the optimal for the highest levels of intracellular ROS production (data not shown). We noted a dose dependent increase in the levels of DCF fluorescence after exposure to the low toxicity nanomaterials (TiO_2_ and MWCNT NMs - Figure 2a, e, f, g, h and j). Following exposure to the highly toxic ZnO (NM 110) and Ag (NM 300) NMs, there was no significant increase in intracellular ROS levels. After exposure of the cells to coated ZnO (NM 111) there was a small but significant increase of fluorescence up to the LC_50_ value before a sharp drop at the higher concentrations. The levels of DCF fluorescence after the exposure to the highly toxic NMs were markedly lower than those witnessed after exposure to the low toxicity nanomaterials (Figure 2b, c and d).

**Figure 3:**
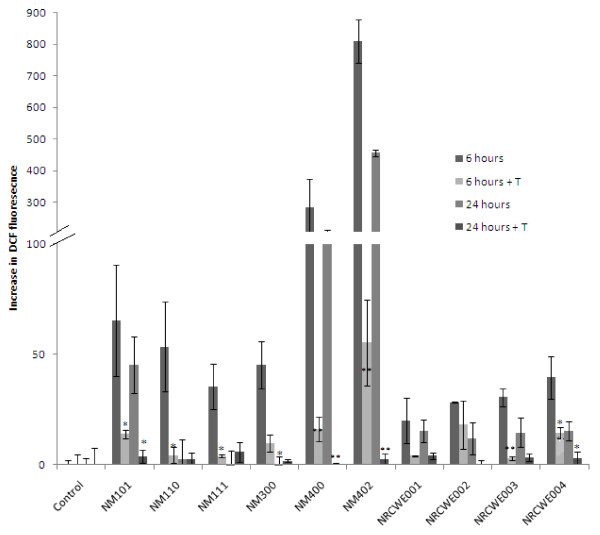
**Effect of 20 μg/cm**^**2**^**of ENPRA nanomaterials on the oxidation of DCFH to DCF in C3A cells with and without Trolox pre-treatment.** The cells were exposed to cell medium (control) or NMs for 6 or 24 hr. Results are exposed as mean fluorescence intensity minus corresponding control traces (±SEM) from three experiments (n = 3), significance indicated by * = *p* < 0.05 and ** = *p* < 0.005, when decrease in fluorescence is compared to cells not treated with Trolox (T) before exposure to the NMs.

In order to investigate whether an antioxidant could prevent the NM-induced ROS production within the hepatocytes – the cells were pre-treated with the vitamin E derivative – Trolox for 1 hr before the addition of the nanomaterials. Trolox prevented the NM induced DCF fluorescence with the inhibition most evident for the two MWCNTs (Figure 3).

### The effect of Trolox pre-treatment on cytotoxicity and interleukin 8 (IL8) production from C3A cells

In a previous study we showed that three of the ENPRA panel of nanomaterials were highly cytotoxic (Ag, coated and uncoated ZnO). We also witnessed an increase in levels of IL8 production following exposure to the NMs investigated while no change in the levels of IL6, TNF-α or C reactive protein was witnessed [17]. In this study we aimed to investigate the effects of the pre-treatment with an external antioxidant on cytotoxicity and IL8 production from the cells. The C3A hepatocytess were pre-treated with Trolox for 1 hr before being exposed to four of the highest concentrations of the nanomaterials used in our previous study (5 μg/cm^2^ to 80 μg/cm^2^).

**Figure 4:**
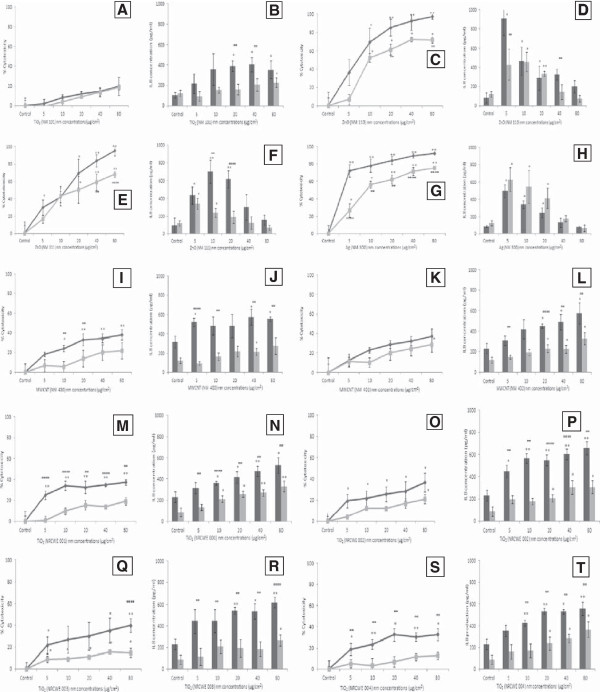
**The cell viability and induction of IL8 production in C3A cells treated with the ENPRA NMs for 24 hr.** Cells pre-treated with the antioxidant Trolox (100 μM, 1 hr) are shown in grey, while cells not pre-treated are shown in black. Values represent mean ± SEM (n = 3), significance indicated by * = *p* < 0.05 and ** = *p* < 0.005, when nanomaterial treatments are compared to the control. ∞ = *p* < 0.05 and ∞∞ *p* < 0.005 is representative of significant difference between values signifying absence and presence of Trolox pre-treatment at each given concentration. **A**) NM 101 cytotoxicity **B**) NM 101 IL8 secretion **C**) NM 110 cytotoxicity **D**) NM 110 IL8 secretion **E**) NM 111 cytotoxicity **F**) NM 111 IL8 secretion **G**) NM 300 cytotoxicity **H**) 300 IL8 secretion **I**) NM 400 cytotoxicity **J**) NM 400 IL8 secretion **K**) NM 402 cytotoxicity **L**) NM 402 IL8 secretion **M**) NRCWE 001 cytotoxicity **N**) NRCWE 001 IL8 secretion **O**) NRCWE 002 cytotoxicity **P**) NRCWE 002 IL8 secretion **Q**) NRCWE 003 cytotoxicity **R**) NRCWE 003 IL8 secretion **S**) NRCWE 004 cytotoxicity **T**) NRCWE 004 IL8 secretion.

We found that pre-treatment with Trolox prevented the cytotoxicity induced by five of the ten nanomaterials investigated (NM 110 (ZnO uncoated), NM 111 (ZnO coated), NM 300 (Ag), NRCWE 001 (TiO_2_ rutile 10 nm) and NRCWE 004 (TiO_2_ rutile 94 nm)) (Figure 4c, e, g, m and s). We also observed that Trolox reduced IL8 secretion for all NMs with the exception of Ag, wherere IL8 secretion appeared increased, but these changes were not significant (Figure 4h).

### DNA damage in C3A cells

In order to investigate the possible DNA damage caused by the panel of nanomaterials, C3A cells were exposed to the NMs for 4 hr. In this study we chose the LC_20_ value for each individual NM plus one concentration above (2x LC_20_) and one below (0.5x LC_20_) (The LC_50_ and LC_20_ values have been previously described) [17].

**Figure 5:**
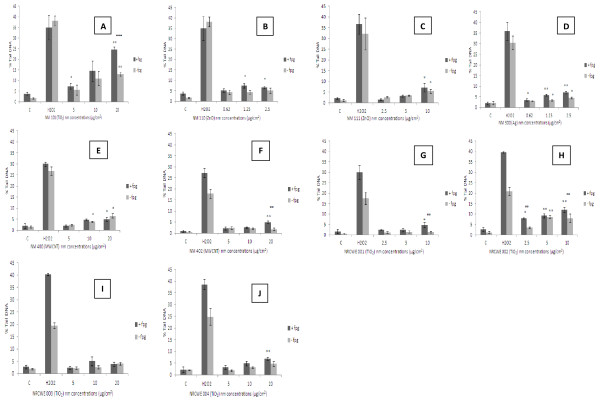
**DNA damage expressed as percent of tail DNA following exposure of the C3A cells to LC**_**20**_ **± one serial dilution to the ENPRA panel of engineered nanomaterials.** The cells were exposed to cell medium (control), 60 μM H_2_O_2_ and NMs for 4 hr. Values represent mean ± SEM (n = 3), significance indicated by * = *p* < 0.05 and ** = *p* < 0.005, when material treatments are compared to the control. ∞ = *p* < 0.05 and ∞∞ *p* < 0.005 is representative of significant difference between values signifying absence and presence of FPG enzyme at each given concentration.** A**) NM 101 **B**) NM 110 **C**) NM 111 **D**) NM 300 **E**) NM 400 **F**) NM 402 **G**) NRCWE 001 **H**) NRCWE 002 **I**) NRCWE 003 **J**) NRCWE 004.

We observed that DNA damage was most evident following exposure to NM 101 (TiO_2_ - 7 nm) and NRCWE 002 (TiO_2_ - 10 nm positively charged) (Figure 5a, h). We also noted a small but significant increase in percentage tail DNA following exposure to seven of the other eight NMs investigated (NRCWE 003 - negatively charged TiO_2_ 10 nm being the exception) (Figure 5).

Long-term 8 week NM 300 (Ag) exposed cells had a small but significant increase in tail moment compared to our cell only control, however there was no significant difference between short and long term exposure to LC_20_ of Ag (data not shown).

Addition of the FPG enzyme to the samples resulted in increased percentage of tail DNA following treatment with the NMs. This indicates that the damage witnessed is partially due to oxidative DNA damage.

## Discussion

This study was conducted as part of a large consortium (FP7 project – ENPRA) to investigate the potential hazard of a wide range of nanomaterials on a variety of targets in order to use the data for generation of a structure activity relationship and for modelling risk assessment. For this reason, the wide dose response ranges were used in order to allow calculation of values such as LC_50_ for comparisons between different materials and cell target types both *in vitro* and *in vivo*. Our previous studies have demonstrated that the panel of nanomaterials investigated could be divided into a high toxicity group (two ZnO and one Ag) and a low toxicity group (five TiO_2_ and two MWCNTs) according to their ability to induce cytotoxicity in the C3A cell line [17] primary human hepatocytes [Kermanizadeh, *et al*., 2012 – Nanotoxicology in press] and primary rat hepatocytes [Filippi, *et al*., manuscript in preparation]. The discussion below is therefore structured to allow comparison between the low toxicity and high toxicity materials in order to ascertain whether this pattern is retained across a wider array of sub-lethal endpoints.

Investigated nanomaterials were characterized by a combination of analytical techniques in order to infer primary physical and chemical properties useful to understand their toxicological behaviour (Table 1) [17]. In order to investigate how the panels of nanomaterials behaved in complete MEM, the hydrodynamic size distributions of the NMs was measured by DLS after the nanomaterials were dispersed in the complete medium between 1–128 μg/ml [17]. Furthermore the 24 hr dissolution of ZnO (NM 110 and NM 111) and the Ag NMs (NM 300) was investigated in the complete C3A medium using atomic absorption spectroscopy. We found that the two ZnO NMs were highly soluble in the medium while the amount of dissolved Ag was very low [17].

Both of the materials with significant solubility are in the high toxicity group while the low solubility nanomaterials are in the low toxicity group. This could lead to the conclusion that increased solubility leads to higher toxicity. However, this is too general as a conclusion, as real toxicity is dependent on the chemical element in question (Table 2).

**Table 2:** Summary of the observed effects on C3A hepatocytes following exposure to the ENPRA panel of nanomaterials

**NM**	**LC50 (WST-1)**	**GSH Depletion**	**Increase in intracellular ROS levels**	**DNA damage**
**NM 101**	> 80 μg/cm^2^	No	Large	Yes
**NM 110**	5 - 10 μg/cm^2^	Yes	None	Yes
**NM 111**	10 - 20 μg/cm^2^	Yes	Small	Yes
**NM 300**	1.25 - 2.5 μg/cm^2^	Yes	None	Yes
**NM 400**	> 80 μg/cm^2^	Yes	Large	Yes
**NM 402**	> 80 μg/cm^2^	Yes	Large	Yes
**NRCWE 001**	> 80 μg/cm^2^	No	Large	Yes
**NRCWE 002**	> 80 μg/cm^2^	No	Large	Yes
**NRCWE 003**	> 80 μg/cm^2^	No	Large	No
**NRCWE 004**	> 80 μg/cm^2^	No	Large	Yes

Initially we tested the ability of the chosen NMs to induce glutathione depletion in C3A cells. Exposure to the Ag and the two ZnO NMs resulted in significant GSH depletion following a 24 hr exposure. For these highly toxic NMs glutathione depletion at the higher concentrations might be associated with cell death rather than a specific oxidative stress response. In contrast, MWCNTs were also able to significantly deplete glutathione at doses which did not influence cell viability, suggesting that MWCNTs induced oxidative stress in these cells. The TiO_2_ materials had no significant effect on the glutathione content of the cells suggesting that they are unable to induce oxidative stress in liver cells in dark experimental conditions.

Next, we investigated intracellular ROS levels following exposure of the ENPRA panel of NMs. Interestingly the Ag and uncoated ZnO NMs did not generate detectable intracellular ROS according to the DCFH-DA assay at 24 hr, although small yet significant levels could be detected for coated ZnO (NM 111). This assay is dependent on intact viable cells therefore exposure to highly toxic nanomaterials could result in lower levels of fluorescence from the DCFH-DA assay. Furthermore, investigations into earlier exposure time-points (6 hr) revealed higher levels of DCF fluorescence for all of the NMs tested. We therefore suggest that the DCFH assay is not suitable for investigating the cellular ROS response to highly toxic NMs, or that it should be limited to relatively low concentrations and early time points for such materials.

In contrast we were able to observe a concentration dependant increase in ROS levels following exposure to the low toxicity nanomaterials (five TiO_2_ and two MWCNT NMs) indicating firstly that these low toxicity materials are able to generate intracellular ROS and secondly that the assay is suitable for these lower toxicity NMs. It is interesting to note that the ROS production by MWCNT translated into a GSH depletion at 24 hr, but the same was not true for the TiO_2_ NMs. Either the cells were sufficiently protected with antioxidant defence mechanisms to prevent GSH depletion by the TiO_2_ NM, or a longer time point might be required to assess such an effect. Another explanation for the ROS generation by the MWCNT might be that the iron residues within the nanotubes may contribute to the oxygen species generation (e.g. via Fe^2+^ fenton reaction).

A recent study investigated a rat derived liver cell line (BRL 3A) and 10 nm Ag NMs (up to 50 μg/ml–24 hr exposure) and reported a significant GSH depletion [23]. In a contradictory study however the effects of silver NPs (220 μg/ml) on primary mice hepatocytes *in vitro* revealed a small increase in intracellular GSH levels subsequent to a 24 hr exposure to the particles [24]. Studies in which human Chang liver cells were exposed to Ag NPs also induced intracellular ROS generation [25] which was contrary to findings in this study. However it is important to note that these finding were after much shorter exposure times [25] which could therefore mean that viability of these cells was sufficient to allow ROS to be detected. Exposure of HepG2 cell to a 100 nm ZnO NMs resulted in high toxicity associated with reactive oxygen species and oxidative stress [26].

We could not identify any studies in which the impacts of MWCNTs on hepatocytes was reported, but intraperitoneal injection of functionalized SWCNTs into Swiss-Webster mice resulted in increased ROS levels within liver cells and enhanced the activities of serum amino-transferases [27].

In a recent set of trials the use of nanoparticulate TiO_2_ (intragastric administration) resulted in mice liver damage with the authors suggesting oxidative stress as the mechanism of cytotoxicity [28]. In another study exposure of mice (intragastric administration) to TiO_2_ NMs for 60 days resulted in hepatocyte apoptosis associated by increased reactive oxygen species accumulation and decreased stress-related gene expression levels of superoxide dismutase and glutathione peroxidise [29]. These studies add support to our observations for MWCNT and TiO_2_ with respect to ROS production in liver cells.

The lipophilic antioxidant Trolox decreased the Ag and ZnO induced cytotoxicity as well as the ZnO induced IL8 production. With respect to Ag, Trolox appeared to enhance IL8 production although this was not statistically significant. This might seem counter-intuitive, as it could be interpreted as protection of the cells from particle-induced cytotoxicity by the antioxidant, thereby enhancing their ability to induce a pro-inflammatory response at doses that were toxic in previous experiments without the addition of Trolox [17]. The pre-treatment of the C3A cells with Trolox prevented the low toxicity nanomaterials from increasing DCF fluorescence, confirming that the DCFH assay was measuring oxidative activity. The pre-treatment with Trolox also resulted in protection against cytotoxicity following exposure to relatively high concentrations of TiO_2_ NMs (NRCWE 001, NRCWE 004). In addition, Trolox pre-treatment decreased the IL8 secretion following exposure to these NMs. Taken together these results suggest that ROS play a key role in the up-regulation of cytokines in hepatocytes following exposure to ZnO, TiO_2_ and the MWCNT NMs.

To our knowledge there have been no nanotoxicological studies that have pre-treated liver cells with Trolox *in vitro*, however Trolox pre-treatment of human macrophages significantly reduced the toxicity of superparamagnetic iron oxide [30] and TiO_2_ NMs [31]. The authors of two other studies in which human monocytes were pre-treated with Trolox before exposure to fine carbon black also noted a decrease in the pro inflammatory cytokine TNF-α [14,32]. Contrary to these findings, pre-treatment with Trolox of J774.A1 macrophages [33], PC 12 cells (cell line derived from a pheochromocytoma of the rat adrenal medulla) [34], N9 (murine microglial cell line) [34] followed by exposure to quantum dots did not prevent toxicity or cytokine production by the cells.

We also investigated any possible genotoxic effects following exposure to the NMs at sub-lethal concentrations. Short term exposure (4 hr) of C3A cells to the ENPRA panel of nanomaterials resulted in a small but significant increase in percent tail DNA for nine of the ten NMs investigated (the negatively charged TiO_2_ - NRCWE 003 being the exception). Exposure of the C3A cells to LC_20_ of Ag NM for 8 weeks resulted in a marginal yet significant increase in tail moment compared to the control, however there was no significant difference between short and long term exposure to this particular particle. We also noted a small but significant increase in DNA damage following exposure to both ZnO and the two MWCNT NMs. Genotoxicity was most evident following exposure to NM 101 (TiO_2_ 7 nm) and NRCWE 002 (positively charged TiO_2_ 10 nm). The relative genotoxicity of the particle panel is therefore strikingly different to their ranking with respect to cytotoxicity. This therefore indicates the importance of assessing sub-lethal effects and the need for further chronic *in vivo* studies to assess the validity of these short term *in vitro* observations.

It is important to emphasis the role of FPG enzyme in the comet assay. The enzyme measures specific oxidative DNA mediated strand breaks so it is not surprising to see increased tail length in the presence of the enzyme following exposure to one of the MWCNTs (NM 402) and three of the TiO_2_ NMs (NM 101, NRCWE 001 and NRCWE 002). As seen from the data in the DCFH-DA assay there was significant increase in intracellular ROS following exposure of the hepatocytes to these materials. Therefore these findings suggest that ROS plays an important role in the genotoxicity witnessed for the MWCNTs and TiO_2_ NM investigated in this study.

In a recent study exposure of human epidermal cell line A431 to TiO_2_ resulted in significant oxidative stress related DNA damage [35]. Our short-term exposure findings are similar in part to a study in which low concentration exposures of 90 nm TiO_2_ NMs to human embryo hepatocytes did not induce DNA breaks or chromosome damage [36]. Another study using A549 cells alveolar epithelial cells, HepG2 hepatocytes and NRK-52E kidney cells were exposed to a panel of NMs including TiO_2_, Al_2_O_3_, gold and MWCNTs discovered that genotoxicity was weak and that DNA damage was limited to single-strand beaks and/or alkali-labile sites [37].

In conclusion, utilizing this particular *in vitro* hepatocyte model showed that the NM which induced a low cytotoxicity (TiO_2_ and MWCNTs) in our previous study [17] generated intracellular ROS, induced oxidative stress (GSH depletion), and that an oxidative mechanism was involved in both the induction of IL8 protein production and genotoxicity according to the Comet assay. The highly toxic Ag and ZnO NMs appeared to work by different mechanisms. Silver did not generate ROS measurable by the DCFH-DA assay, although pre-treatment with an antioxidant may marginally enhance IL8 production by the hepatocytes suggesting that the toxic mechanisms might be partially mediated by ROS. In addition the data indicates that Ag particles are capable of enhancing a pro-inflammatory response, providing that they are not too toxic. A good understanding of the dissolution kinetics of the nanomaterials during the preparation steps and in the cell mediums are crucial in evaluating of the toxicology of these materials. In a previous publication [17] we have shown that less than 1% of Ag (NM 300) dissolves in this medium after 24 hr of incubation, so it is very unlikely that the damage to the cells is due totally to the release of Ag ions. The ZnO NMs were highly soluble in the C3A medium so there is a real possibility that the high toxicity of these particles is in part due to the release of ions.

Future studies will concentrate on co-culture of primary rat liver cells with phagocytic cells (hepatocytes and Kupffer cells) with particular attention on cytotoxicity and cytokine production as well as trying to ascertaining the potential mechanism driving inflammation in particular the role of reactive oxygen. Studies conducted by project partners will employ other target cells such as macrophages, lung epithelial cells, fibroblasts, endothelial cells and renal proximal tubule epithelial cells. *In vivo* studies are also being conducted for comparison with *in vitro* models. All of this data will be combined into a database to be used for a structure activity relationship and for risk assessment modelling.

## Competing interests

The authors declare that they are no competing interests.

## Authors’ contributions

AK has carried out the experiments within this study. BKG, GRH and VS have all been heavily involved in the preparation and revision of the manuscript. All authors have read and approved the final manuscript.

## Funding

This work has been financially supported by the European seventh framework programme co-operation [Grant code – NMP4-SL-2009-228789].
